# Differential Host Response, Rather Than Early Viral Replication Efficiency, Correlates with Pathogenicity Caused by Influenza Viruses

**DOI:** 10.1371/journal.pone.0074863

**Published:** 2013-09-20

**Authors:** Peter S. Askovich, Catherine J. Sanders, Carrie M. Rosenberger, Alan H. Diercks, Pradyot Dash, Garnet Navarro, Peter Vogel, Peter C. Doherty, Paul G. Thomas, Alan Aderem

**Affiliations:** 1 Seattle Biomedical Research Institute, Seattle, Washington, United States of America; 2 Department of Immunology, St. Jude Children’s Research Hospital, Memphis, Tennessee, United States of America; University of Edinburgh, United Kingdom

## Abstract

Influenza viruses exhibit large, strain-dependent differences in pathogenicity in mammalian hosts. Although the characteristics of severe disease, including uncontrolled viral replication, infection of the lower airway, and highly inflammatory cytokine responses have been extensively documented, the specific virulence mechanisms that distinguish highly pathogenic strains remain elusive. In this study, we focused on the early events in influenza infection, measuring the growth rate of three strains of varying pathogenicity in the mouse airway epithelium and simultaneously examining the global host transcriptional response over the first 24 hours. Although all strains replicated equally rapidly over the first viral life-cycle, their growth rates in both lung and tracheal tissue strongly diverged at later times, resulting in nearly 10-fold differences in viral load by 24 hours following infection. We identified separate networks of genes in both the lung and tracheal tissues whose rapid up-regulation at early time points by specific strains correlated with a reduced viral replication rate of those strains. The set of early-induced genes in the lung that led to viral growth restriction is enriched for both NF-κB binding site motifs and members of the TREM1 and IL-17 signaling pathways, suggesting that rapid, NF-κB –mediated activation of these pathways may contribute to control of viral replication. Because influenza infection extending into the lung generally results in severe disease, early activation of these pathways may be one factor distinguishing high- and low-pathogenicity strains.

## Introduction

Despite intense research efforts, infection with influenza virus remains a significant source of morbidity and mortality world-wide. Globally, seasonal influenza strains infect three to five million people each year resulting in approximately 250,000 to 500,000 deaths [1]. The economic burden of seasonal influenza in the United States is estimated to exceed $80 billion annually [2,3]. In addition to yearly epidemics, influenza A viruses cause occasional pandemics when a novel strain emerges and the majority of the human population has no immunity. The 1918 influenza pandemic, which killed between 50-100 million people world-wide, was one of the most deadly events in human history [4].

In general, transmission and pathogenicity are uncoupled for the influenza viruses. Although much less virulent than the 1918 virus, the pandemic strains from 1957, 1968, and 2009 transmitted rapidly through the human population [5,6]. In contrast, although mortality rates of up to 60% have been described in infections with avian-origin H5N1 strains, sustained human-to-human transmission has not been reported [7-10]. While seasonal influenza strains readily infect the upper regions of the human respiratory tract, the H5N1 viruses only establish when they penetrate more deeply, likely due to the binding specificity of their hemagglutinin molecules for α2,3-linked sialic acids which are found only in the lung [11-13]. Infection of the bronchi and alveoli, either directly by avian viruses or by seasonal strains that spread from the upper respiratory tract, is highly correlated with severe disease [14-16].

Studies in mice have shown that many genes are involved in the control of influenza virus replication [17,18], with most being induced by type I or type III interferons. While mice entirely lacking both type I and type III interferon responses (IFNAR^-/-^IL28R^-/-^ DKO) exhibit unrestrained viral growth and quickly succumb to disease [19], the ablation of individual cytokines or effectors has a much smaller impact [20-23]. Although these studies suggest that numerous genes contribute cooperatively, and to some extent redundantly, to control infection, determining their relative contributions to host defense remains an area of active research. Furthermore, the degree to which differences in the activation of innate immune responses contribute to influenza pathogenicity, especially very early after infection, has received less attention. Most *in vivo* gene-expression studies aimed at identifying the specific responses that contribute to the innate immune control of influenza have examined time points from 1-7 days after infection, corresponding to many replication cycles of the virus [24-26]. Due to numerous factors, including complex cytokine and chemokine feedback mechanisms and the recruitment of immune cells to the airway, the combined transcriptional profile of infected tissues is difficult to interpret as the disease progresses, and many of the earliest responses to the virus are likely to be obscured.

The present analysis thus focuses on characterizing the innate immune responses that distinguish pathogenic from non-pathogenic strains of the virus within the first 24 hours of lung infection. We targeted this interval in order to uncover differences in the host response directly attributable to virus replication and virulence, while minimizing confounding secondary effects from the divergent course of disease and the involvement of adaptive immunity at later times. The analysis compares the transcriptional response in the lung and trachea following infection with three strains of influenza that span orders of magnitude in pathogenicity (as measured by LD_50_), though they differ only in their surface hemagglutinin and neuraminidase surface glycoproteins.

## Methods

### Viruses

The PR8, X31, and VN1203(6+2) influenza A viruses were generated using the 8-plasmid influenza reverse genetics system [27]. All three viruses are based on PR8 (A/Puerto Rico/8/34). In detail, PR8 is a mouse-adapted H1N1 strain originally derived from a human isolate. X31, is a mouse adapted H3N2 strain with the 6 internal genes of PR8 and the HA and NA derived genes from A/Aichi/2/1968. rgVN1203(6+2) (VN) contains the 6 internal genes of PR8 and the H5N1 HA and NA genes from A/Vietnam/1203/2004 with the polybasic cleavage site of the HA modified to restrict its replication to the airway [28,29]. Virus stocks used in this study have been grown in eggs and tested for microbial contamination by streaking on blood agar plates.

### Mice and infections

Eight to twelve week old female C57Bl/6 mice (Charles River) were anesthetized (Avertin) and infected intranasally (i.n.) with 1x10^5^ PFU of PR8, X31, or VN in 30 μL of PBS or mock-infected with 30 μL PBS alone. At specified time points, mice were sacrificed by CO_2_ inhalation. The right lung and trachea were removed and separately homogenized in TRIzol for 150 seconds using a TissueLyserII (Qiagen, Valencia, CA). The left lung was fixed and used for histology. All mice were cared for under specific pathogen-free conditions in an approved animal facility at SJCRH.

### Ethic statement

All animal work was reviewed and approved by the appropriate institutional animal care and use committee at SJCRH (protocol #098) following guidelines established by the Institute of Laboratory Animal Resources and approved by the Governing Board of the U.S. National Research Council.

### Cells

The LET-1 cell lines were generated from female C57Bl/6 or IFNAR^-/-^ mouse type I lung epithelial cells transformed with large-T antigen [C. Rosenberger and V. Tam – unpublished data]. All animal work for the generation of the LET-1 cell line was performed in accordance with approved Institute for Systems Biology IACUC protocols. LET-1 cells were cultured in Dulbecco’s Modified Eagle Medium (DMEM), containing 2 mM L-glutamine, penicillin, streptomycin, and 10% FBS. The alveolar macrophage cell line MH-S (ATCC CRL-2019) and the dendritic cell line JAWSII (ATCC CRL-11904) were obtained from ATCC and cultured as recommended. Primary mouse tracheal epithelial cell (mTEC) cultures were prepared with a modified protocol described previously in [30].

### *In vitro* infection of mTEC cultures

The exposed apical surface of the ciliated, polarized pseudo-stratified mTEC cultures was washed four times with pre-warmed HBSS and moved to new 24 well plates prior to infection. The basolateral media was replaced with 1mL of pre-warmed Differentiation Medium (DM) [30]. Viruses were added in 200 μL DM to the apical side only of these transwell cultures, which were then incubated at 37°C for 1 hour. Then the inoculum was removed, the cultures were washed once with pre-warmed HBSS and the apical surface was maintained dry (sustained across the membrane by the medium below) at 37°C. At time of harvesting, the exposed surfaces were washed with 250μL DM twice, the basolateral medium was removed, and 250μLTRIzol (Invitrogen) was added to the apical side. The cells were scraped free with a p1000 pipet tip, and the wells were rinsed with an additional 250μL of TRIzol.

### LET-1, MH-S, JAWSII and MLE 12 cells

Cells were seeded into 24-well plates at 2x10^5^ cells per well (BD Falcon) and allowed to adhere overnight at 37°C + 5% CO_2_. The monolayer cultures were then washed with warm phosphate buffered saline (PBS), followed by infection with virus in complete medium for 1 hour. Virus-infected cells were washed three times with warm PBS, then complete medium was added for the duration of infection. For collection, cells were washed once with warm PBS and harvested using 500μL TRIzol.

### Tnf treatment of LET-1 cells

The LET-1 cells were infected as described above. At 1hour after infection, following media replacement, recombinant Tnf (PeproTech Inc. #315-01A) was added at 100 ng/ml and left in the culture through the remainder of the experiment.

### Lung cell isolation and cell sorting

Mice were sacrificed using C0_2_ inhalation. A tracheal cannula was inserted and 1.5mL dispase (BD Bioscience #354235) (5mg/mL) injected, followed by 0.5mL 1% wt/vol agarose (low melting) which was then solidified by packing the lungs with ice for 2 min. The lungs were dissected out and incubated for 45 min in 2mL dispase solution at RT. Lungs were transferred to DMEM with 25mM HEPES containing DNase I (50μg/ml). Dissected lung tissue was disrupted into a single-cell suspension by sequential passage through 100μm, 70μm and 40μm filters (washed with 10ml DMEM 10% FBS + HEPES + P/S + NEAA for each filter). The cells were centrifuged at 350×g for 10 min at RT, resuspended in 1ml ACK and 9ml of DMEM 10% FBS + HEPES + P/S + NEAA, centrifuged at 350×g for 10 min at RT, and resuspended in 2ml FACS buffer. Then 10 μL of 2.4G2 antibodies (UCSF Monoclonal Antibody Core #IMMZC001) was added to block the Fc receptor, the cells were incubated on ice for 10 min, stained for 15 min with a 1:200 dilution of T1a-PE (eBioscience #12-5381) and sorted on the BD FACS ARIA II for T1a+ cells.

### Histology

Left lungs from infected mice were fixed via intratracheal infusion and then immersion in 10% buffered formalin solution. Tissues were paraffin embedded, sectioned, and stained for virus using a primary goat polyclonal antibody (US Biological, Swampscott, MA) against influenza A, USSR (H1N1) at 1:1000 and a secondary biotinylated donkey anti-goat antibody (catalog number sc-2042; Santa Cruz Biotechnology, Santa Cruz, CA) at 1:200 on tissue sections subjected to antigen retrieval for 30 minutes at 98°C. Blinded sections were examined by a pathologist and scored for the distribution of influenza virus antigen on the following scale: 0 = No virus positive cells; 1 = Minimal, focal to multifocal, inconspicuous; 2 = Mild, multifocal, conspicuous; 3 = Moderate, multifocal, prominent lesions; 4 = Marked, multifocal coalescing; 5 = Severe, extensive, diffuse, with consolidation, multilobar.

### Plaque assay

Whole mouse lungs and tracheas were isolated, homogenized in infection medium (0.3% BSA, 0.45% NaHCO3 in MEM), and added to confluent monolayers of MDCK cells in 10-fold dilutions (10^-1^ to 10^-6^) for one hour. These dilutions were removed and replaced with agar containing 2x MEM and 1µg/ml TPCK treated trypsin (both Sigma-Aldrich, St. Louis, MO). After 37°C incubation for 72 hours, agar coatings were removed, cell layers were stained with crystal violet, and plaques were counted for titer calculation.

### Microarray analysis

RNA was extracted from TRIzol (Invitrogen) according to the manufacturer’s recommendations, and cDNA was synthesized from DNase treated RNA (Ambion) and random primers using Invitrogen superscript II (Invitrogen). The RNA was then sent from St Jude to the Institute for Systems Biology for array analysis using the SurePrint G3 Mouse GE 8x60K Microarray Kit. The data discussed in this publication have been deposited in NCBI’s Gene Expression Omnibus [31] and are accessible through GEO Series accession number GSE42285 (http://www.ncbi.nlm.nih.gov/geo/query/acc.cgi?acc=GSE42285).

### Data analysis

Array expression data from Agilent arrays (above) was linearized and loaded into Genedata Analyst (Genedata AG, Basel, Switzerland). All samples were first subject to Quantile normalization and the relative fold change (relative to Mock infected samples) were determined. Genes which had expression levels below the threshold (log_2_ value < 6) and genes which showed high variation in the mock-infected samples (coefficient of variation > 10%) were excluded. ANOVA analysis was performed using the K groups option with 100 balanced permutations. Group medians were used to calculate effect size. Clustering of selected genes was performed using Positive Correlation Distance (1-r) with 1000 max iterations with following settings: centroid calculations: Median, sampling method: bootstrap, sampling percentage: 70 and number of repeats: 100.

### Real-time PCR

Expression of the influenza M-gene was determined by real-time PCR using primers specific for the M1 transcript: forward: 5'-/ TCA GGC CCC CTC AAA GCC GAG AT/-3'; reverse 5'-/CGT CTA CGC TGC AGT CCT C/-3'; probe: 5'-/56-FAM/TTT GTG TTC ACG CTC ACC GTG CCC A/3BHQ_1/-3'. Ef1a primers used were: forward: 5'-/GCA AAA ACG ACC CAC CAA TG/-3'; reverse: 5'-/GGC CTG GAT GGT TCA GGA TA/-3'; probe 5'-/56-FAM/CAC CTG AGC AGT GAA GCC AG/36-TAMSp/-3'

### Microfluidics based quantitative PCR

cDNA was pre-amplified for 14 cycles using a 0.2× mixture of pooled 20× Taqman probe sets (ABI). The PCR was run on the BioMark™ HD System (Fluidigm Corporation, South San Francisco, CA) using 48x48 Dynamic Array IFC (Fluidigm) according to the manufacturer’s instructions.

### Promoter enrichment analysis

For Clover analysis, promoter sequences (1500/500 bp before/after transcription start site) were obtained from an internal database which was populated using sequences obtained from BioMart. The Clover program [32] was compiled and run on a local server using default parameters except for the motif score threshold (7). Two other parameters were modified as needed – “P-value threshold” to obtain all p-values for selected genes and “number of randomizations” to increase the precision (up to 1,000,000). Scanning was performed with motifs from the TRANSFAC 2010 Professional database. Promoter sequences from all genes expressed in the array experiment (log_2_(expression) > 6) were used as a background for the p-value calculations. A complete motif database was initially used with 1000 iterations and motifs enriched with p-Values of 0.001 or better were selected and rerun with a p-Value cutoff of 1 (to get p-values for those motifs for all groups). Separately, a new motif database was created with motifs that had p-values of 0 in any group and the program was rerun against the smaller database with an increased number of iterations. This process was repeated until the number of iterations reached 1,000,0000 for the final set of motifs. The oPOSUM program was run from http://www.cisreg.ca/cgi-bin/oPOSSUM/opossum using default settings [33].

### Pathway enrichment analysis

Data were analyzed through IPA (Ingenuity Pathway Analysis - Ingenuity® Systems). A data set containing gene (or chemical) identifiers and corresponding expression values was uploaded into the application. Each identifier was mapped to its corresponding object in the Ingenuity® Knowledge Base. Canonical pathways analysis identified the pathways from the IPA library of canonical pathways that were most significant to the data set. Fisher’s exact test was used to calculate a p-value for the probability that the association between the genes in the dataset and the canonical pathway is explained by chance alone. A Benjamini -Hochberg corrected p-value ≤ 0.05 was used as the threshold of significance. Pathways containing less than three genes from the set were removed, as were pathways that are not biologically relevant for lung tissue. 

## Results

### Pathogenic influenza viruses replicate to significantly higher titers in the lung

**Figure 1:**
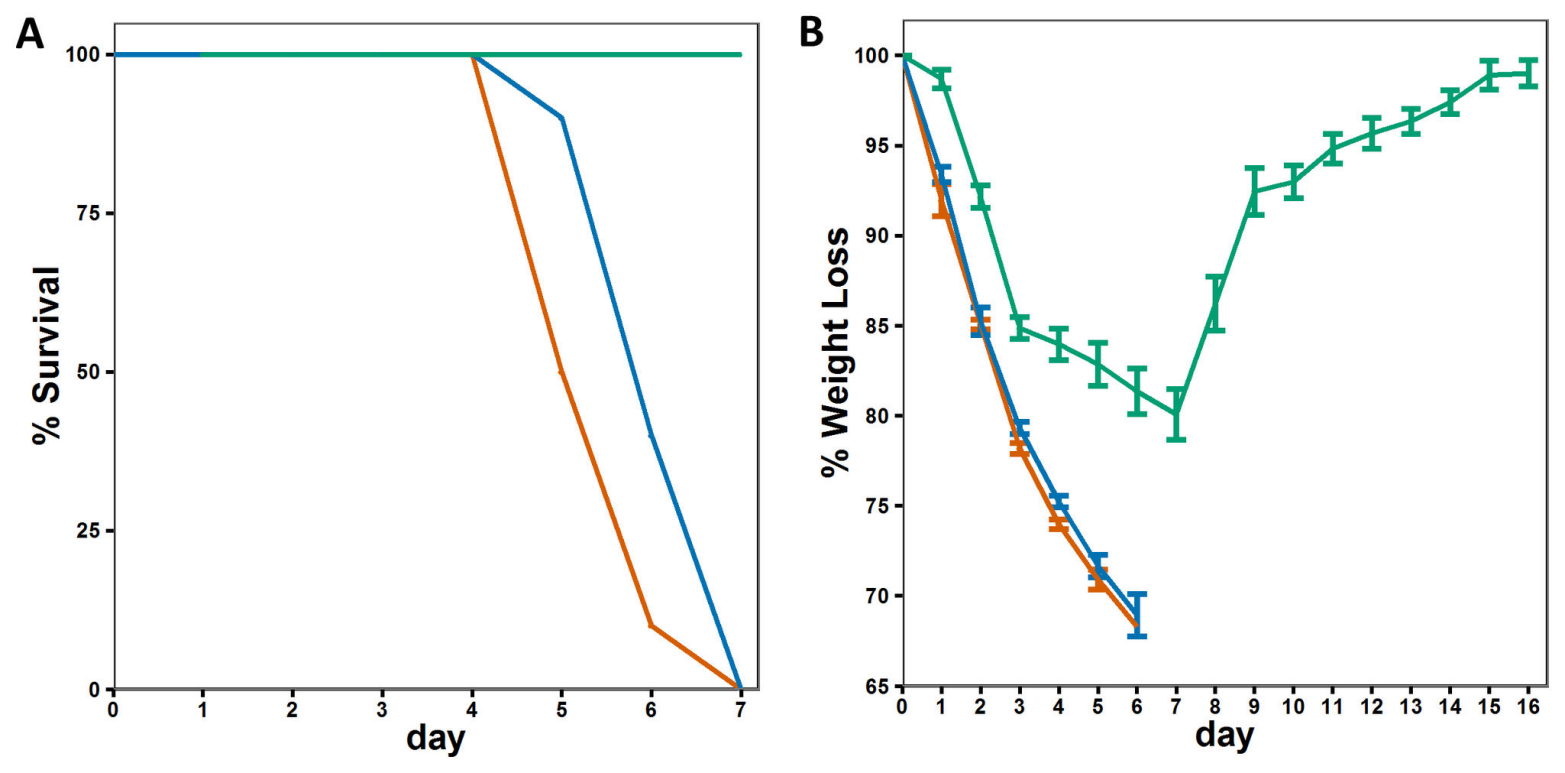
Relative pathogenicity of PR8, X31, and VN. A) Survival and B) weight-loss for mice infected with 10^5^ PFU (red=PR8, green=X31, blue=VN). Error bars indicate the SEM for 10 mice at each time point.

**Figure 2:**
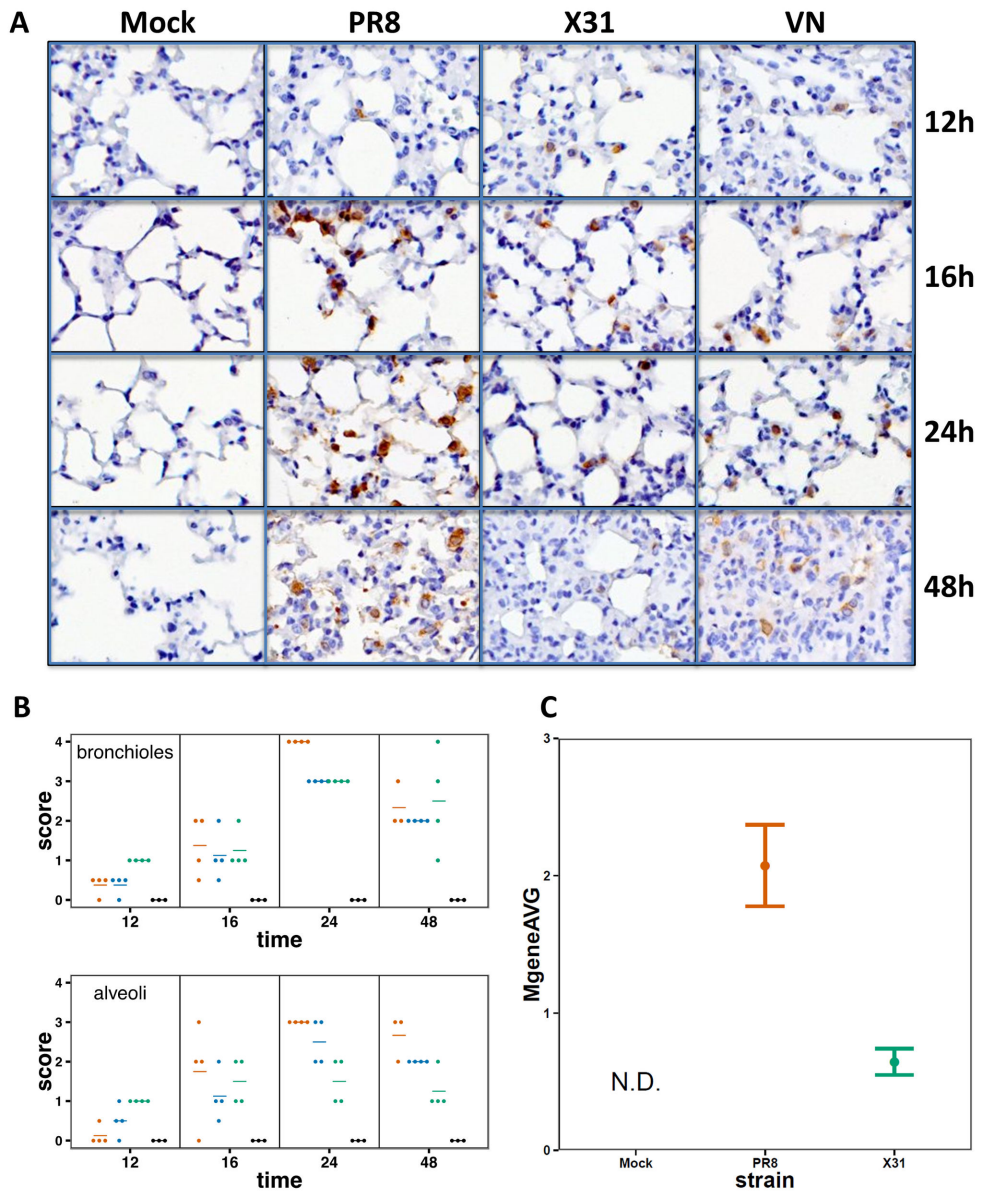
Replication of influenza virus in mouse lungs. A) Immunohistochemistry of influenza-infected lungs. Paraffin embedded sections of mouse lungs were stained using an anti-HA antibody. Tissues were collected at the indicated times. B) Histological scoring of the extent and distribution of infection in the lungs. Each dot represents the score for an individual animal; horizontal bars represent the mean score for the group (red=PR8, green=X31, blue=VN). C) Influenza M-gene expression in T1a+ sorted lung cells 18 hours post-infection. T-test p-value for a difference between X31 and PR8 is 0.0018.

**Figure 3:**
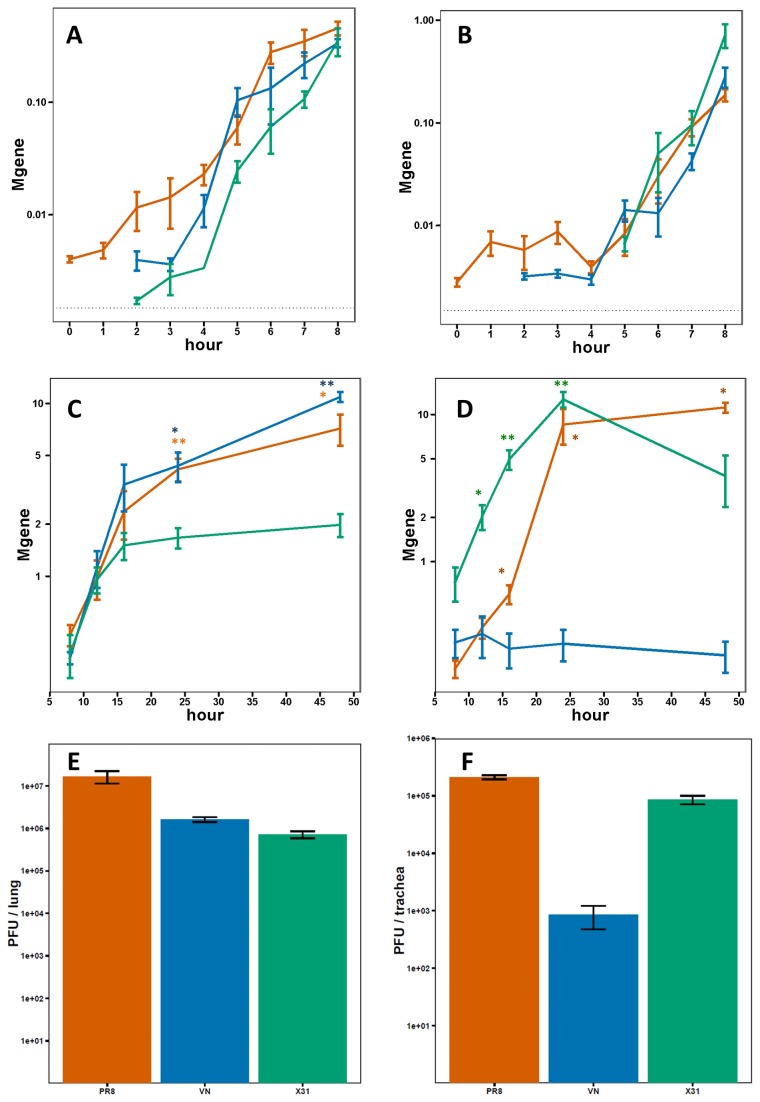
Replication of influenza virus in mouse lungs and trachea. Quantification of viral genomes in the lung and trachea A, B) during the first 8 hours following infection and C,D) from 8 to 48 hours following infection (red=PR8, green=X31, blue=VN). Error bars indicate the SEM for 3-5 mice at each time point. The dotted line indicates the minimum quantifiable level (* p < 0.05, ** p < 0.001 for a non-zero difference in M-gene expression of each strain relative to C). X31 or D) VN). E and F) Viral titer in lungs and trachea of infected mice 24 hours post-infection. Values represent average of 3-5 mice. Error bars show standard error.

The basic aim of this analysis is to correlate the characteristics of virus growth with the host transcriptional profiles that distinguish pathogenic from non-pathogenic infections in the murine respiratory tract. The LD_50_ (PFU) for PR8 and VN is ~10^3^, while that for x31 is ~10^6^. Individual mice were infected intranasally (i.n) with 10^5^ PFU of PR8, VN or x31, a dose that causes approximately 20-25% body weight loss by day 4 with x31 (though all mice recover fully) while those given PR8 or VN typically succumb (Figure 1) by day 5-6 [29,34]. Based on the estimated surface area of the murine airway [35], this dose represents an effective MOI of approximately 0.01. The extent of infection in the lungs was assessed using immunohistochemistry over the first 48 hours following infection. The recruitment of immune cells to the lung is minimal during this period [36], and mice show minimal signs of disease. When paraffin embedded sections from lungs taken at different time points were stained for immunohistochemistry using an anti-HA antibody, infected cells were detected in lung sections of mice infected with all strains. The relative distribution and intensity of anti-HA staining were similar for all 3 viruses at 12 and 16 hours (Figure 2A), but the extent of infection for PR8 and VN then diverged strongly from the X31 profile as evidenced by significantly more viral antigen being present in the lungs of mice infected by the two high pathogenicity strains (Figure 2A). Histological scoring confirmed the observed differences in viral antigen abundance. While the infection levels in the bronchioles were similar in all groups, the alveoli of PR8- and VN-infected mice consistently contained more virus positive cells at later time-points (Figure 2B). We then analyzed viral load in the type I lung epithelial cells that comprise most of the alveolar surface [37-39]. Lungs of mice infected with either X31 or PR8 were collected at 18 hours after infection and cells were dissociated, live-labeled, and FACS sorted using a type I specific antibody (anti-T1a). Consistent with the histological findings (Figure 2A and B), levels of the influenza M gene detected by real-time PCR were approximately four times higher in T1a+ cells isolated from PR8-infected compared to X31-infected lungs (p = 0.0018) (Figure 2C),

In order to more precisely define the course of infection with the different strains, viral loads in separate homogenates of the trachea and right lung were determined at closely spaced intervals over the first 48 hours by measuring the abundance of the influenza M gene. Remarkably, all three strains showed profiles of essentially identical, aggressive replication in the respiratory tract over the first 12 hours with the levels of viral M gene increasing by >1000x (Figure 3 A and C). At 16 hours, however, the replication rates started to diverge with the low pathogenicity X31 lagging behind that of the high pathogenicity PR8 and VN strains. Over the next 32 hours, the quantity of X31 in the lung remained essentially constant while that of PR8 and VN continued to increase, reaching levels approximately 5x higher by 48 hours (Figure 3 C). In the trachea, as in the lung, all three strains replicated equally rapidly over the first 8 hours, with the M gene levels increasing approximately 100x (Figure 3B). From 8 hours onward however, the levels of VN remained constant while PR8 and X31 continued to grow rapidly with M gene levels increasing by more than 20x over the next 12 hours (Figure 3D). Importantly, the viral load measurements in the lungs and tracheas were performed in the same individuals, demonstrating the large, tissue-specific differences in replication rates between these three strains. Viral titers in the tracheas and lungs of infected mice at 24 hours post-infection generally reflected the viral abundance as measured by M-gene qPCR. In the lungs (Figure 3E), the X31 titer was lower than both PR8 and VN (23.3- and 2.3-fold respectively). In the trachea (Figure 3F), the VN titer was lower than both X31 and PR8 (101- and 249-fold respectively).

### Early transcriptional response in the lung

**Figure 4:**
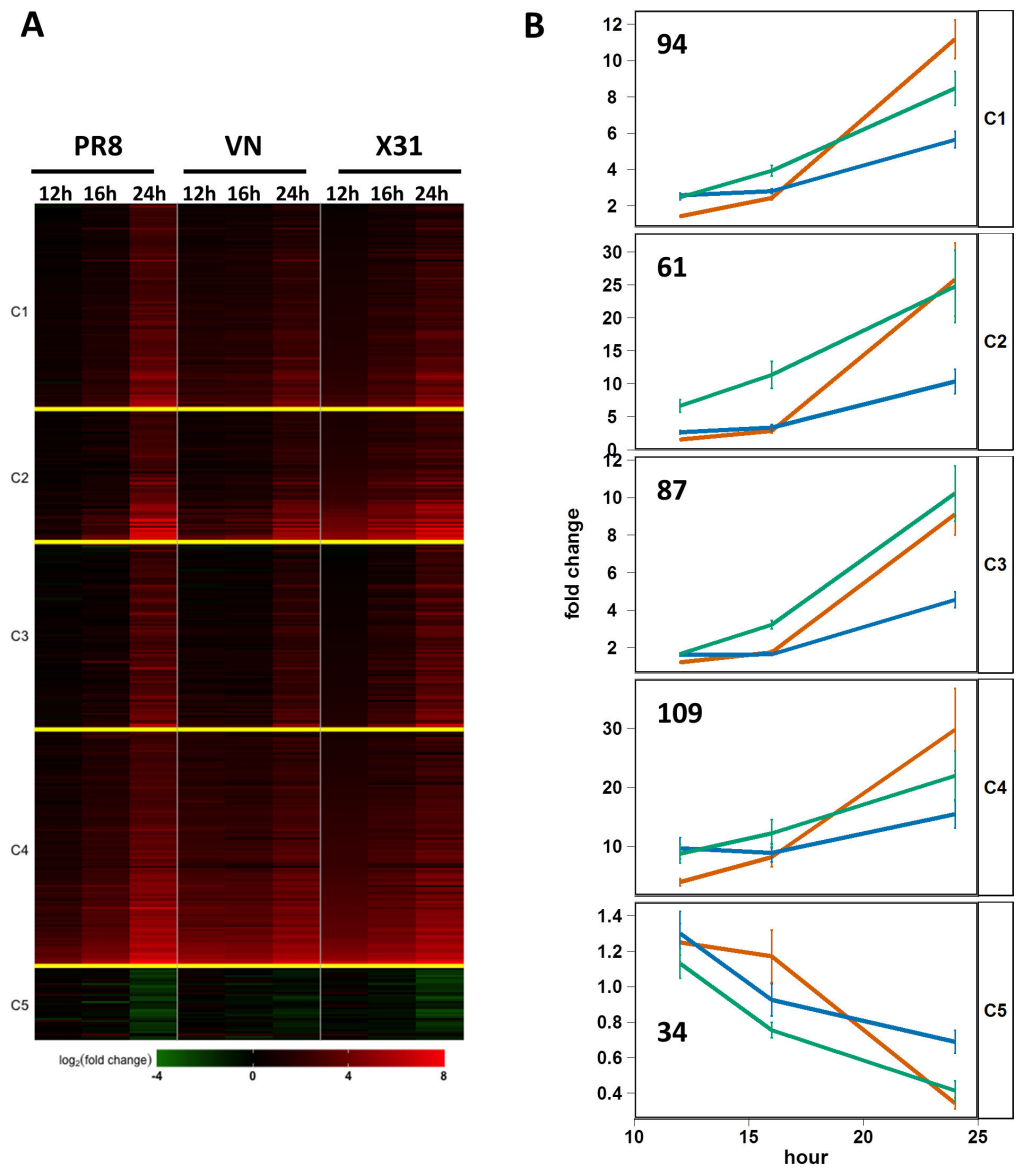
Global expression profiling of lung tissue isolated from influenza-infected mice. A) Heatmap depicting 385 genes whose expression is altered in the lung during the first 24 hours following infection with influenza virus (permutation q-value=<0.001 and > 5-fold difference). B) Mean expression of all genes within each cluster (K means with k=5 for 385 genes) grouped by virus strain (X31 – green; PR8 – red; VN – blue). The number of genes in each cluster is indicated. Error bars depict the SEM for all genes in the cluster at each time point.

The observation that both low- and high-pathogenicity viruses exhibit nearly identical growth kinetics over the first 16 hours was unexpected and suggested that the attenuated growth of X31 after 16 hours (relative to PR8 and VN) either resulted from the induction of a unique host response or from a common response activated with different kinetics. To test this hypothesis, global transcriptome analysis was performed on RNA isolated from whole lung tissue at 12, 16, and 24 hours following infection with the three virus strains. Using a one-way ANOVA and stringent significance cutoffs (permutation q-value=0.001 and at least a 5-fold difference between the highest and lowest groups), we identified 385 genes whose expression was altered by infection for at least one time point (Figure 4A). Stringent cutoffs were used to minimize false positives and focus on the strongest signal. These genes were clustered by their temporal expression profiles using K-means with k=5, which produced a reasonable grouping of similar expression profiles (Figure 4B). This analysis demonstrated several notable patterns of gene expression, some of which distinguished individual virus strains. For example, cluster 2 contains 61 genes that were rapidly up-regulated by challenge with X31, but whose induction following PR8 and VN infections was significantly delayed. A large number of genes within cluster 2 are cytokines, chemokines and related genes, almost all of which are pro-inflammatory (Csf1, Csf2, Il1b, Ccl2, Il12b, Ccl7, Ccl3, Cxcl1, Ccl4, Ccl17, Cxcl9, Il4i1, Socs3, Tnf, Tnfaip3, Tnfsf15, Tnfaip). The remaining genes in this cluster are Rgs16, Timp1, Pip5k1a, Mt1, Inhba, Ptx3, Saa1, Ly6i, Nod2, Ly6f, Cd83, Serpina3h, Serpina3i, Serpine1, 2210406H18Rik, Selp, Irg1, Mcoln2, Tcp10b, Gpr84, Tlr2, Gbp5, LOC637082, AA467197, Tifa, Cfb, Ch25h, Trim15, Lcn2, Slc26a4, Gm6377, LOC100041824, Slc39a14, Rnd1, Bdkrb2, Tcp10c, Pim1, Asb4, Mt2, Arid5a, Noxo1, Casp4, and Gem. Genes in clusters 1 and 4 exhibit similar expression kinetics in all infections, with the two clusters being distinguished by their average magnitude of induction especially at the 12-hour time point. In general, across the entire set of genes and particularly in clusters 2 and 3, the VN strain induces smaller changes in gene-expression than either X31 or PR8.

**Figure 5:**
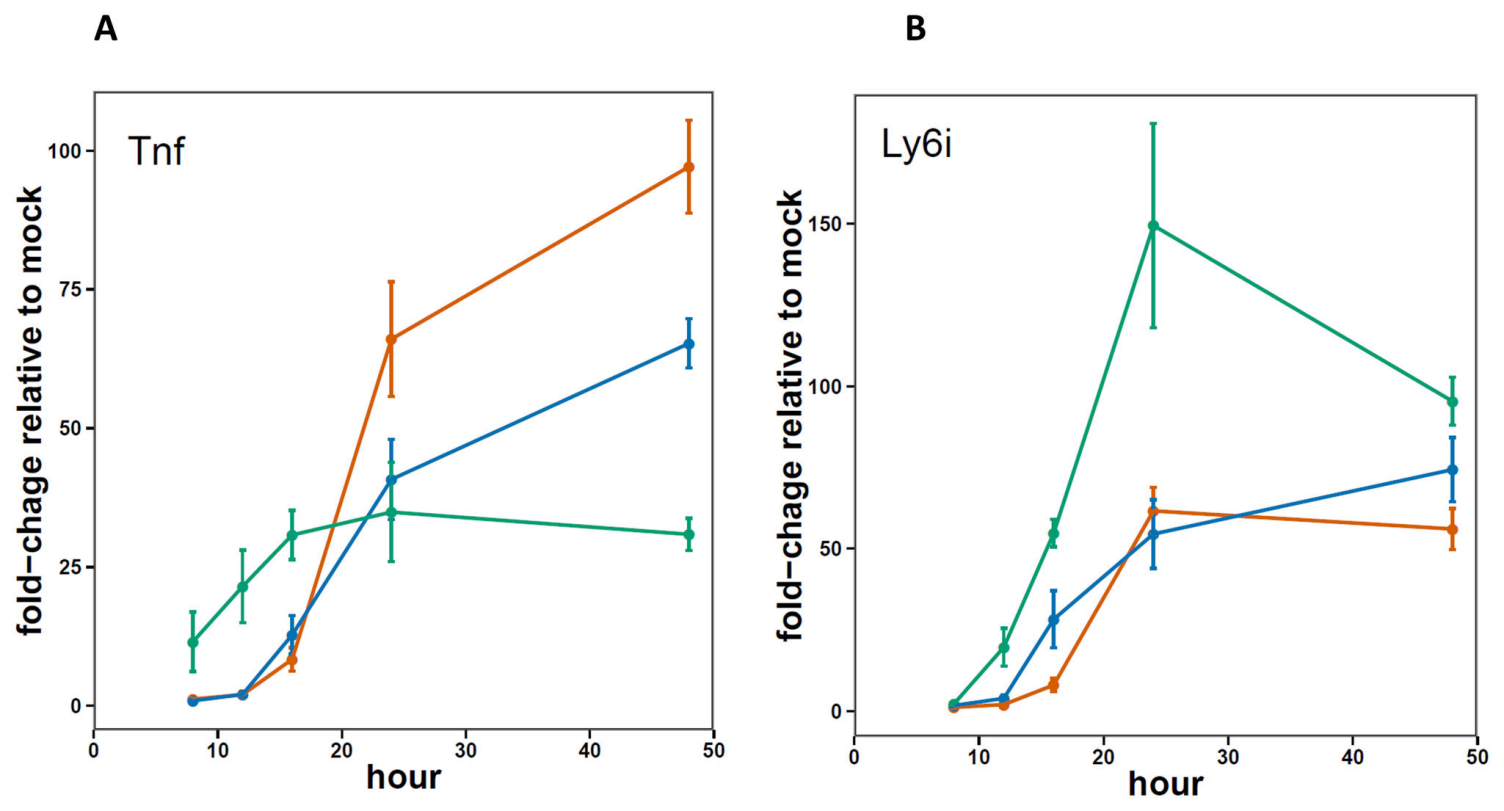
Microfluidics qPCR measurements of Tnf and Ly6i expression in the lungs 8-48 hours post infection. Graphs show average values (red=PR8, green=X31, blue=VN) and error bars depict SEM for 3-5 mice per group.

In order to more precisely define the early host response to influenza infection in mice, RNA isolated from the lungs was analyzed using the BioMark HD System, a microfluidics based qPCR platform (Fluidigm Corporation, South San Francisco, CA). Based on the results of the array analysis, a set of 96 genes was selected to follow throughout the longer time course. Of these 96 genes, 25 were induced more strongly by infection with X31 at either 12 or 16 hours relative to both PR8 and VN (p ≤ 0.05-data not shown). Most of the genes found to increase early in the X31- relative to the PR8 and VN infected mice, were expressed at lower levels at later time points (24 and 48 hours). The exception was the Ly6i gene, which was found to be higher for X31 throughout the experiment (Figure 5 A and B).

### Promoter enrichment analysis

**Figure 6:**
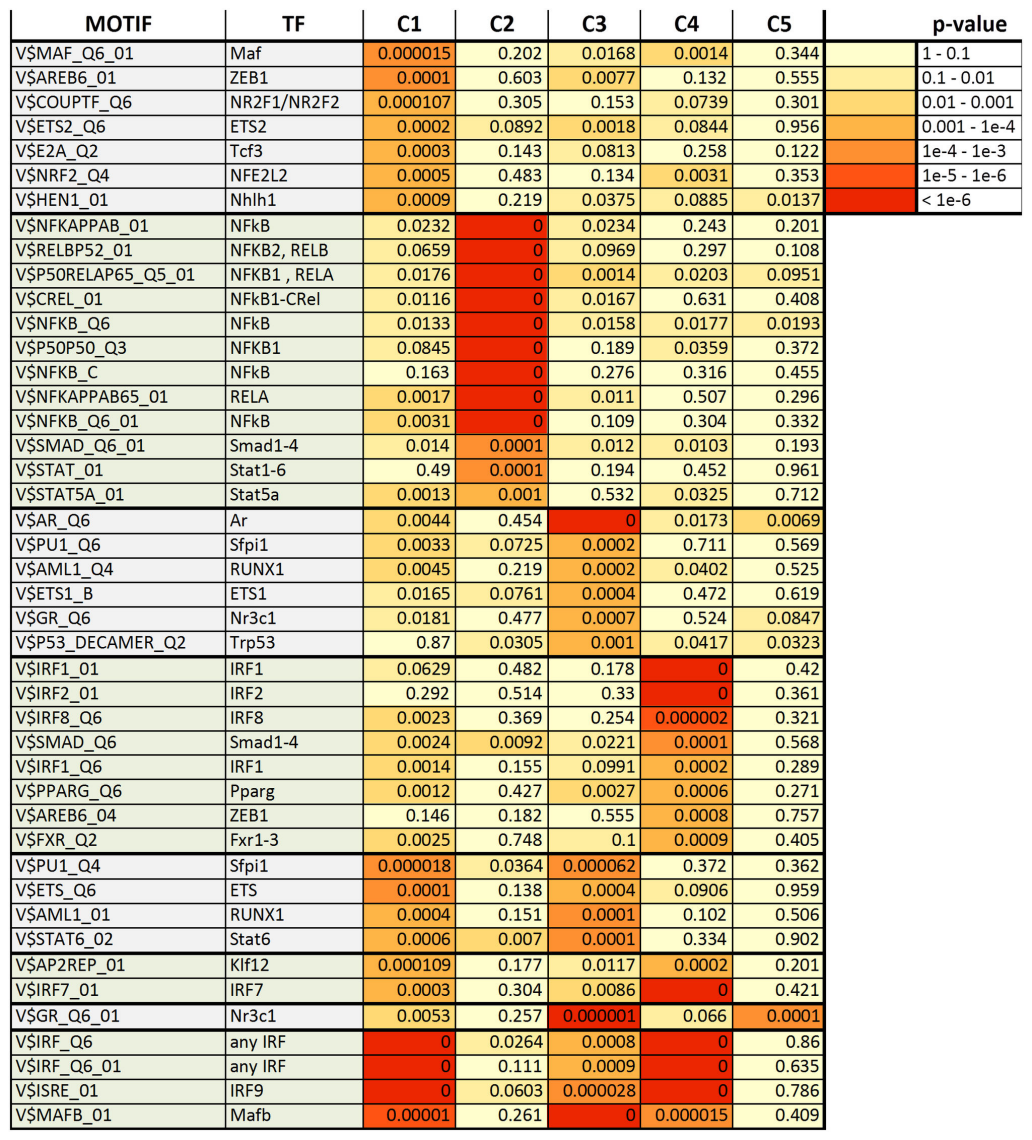
Transcription factor binding site motifs enriched in at least one expression cluster. Enriched motifs (p < 10^-3^) were identified from a Transfac database of 909 unique motifs. Zero (0) indicates motifs enriched at a p-value < 10^-6^, the limiting resolution of the analysis.

In order to identify transcription factors that might specifically regulate each of the expression clusters defined above, the promoter regions (-1500 kb to +500 kb from the transcriptional start site) of each gene were scanned for matches to each of 909 unique binding site motifs (TRANSFAC pro 2010) using CLOVER [32]. Numerous binding site motifs were strongly enriched relative to a background set of genes whose expression was not altered by influenza virus infection (Figure 6). Strikingly, cluster 2 was highly enriched for NF-κB binding site motifs. Enrichment of NF-κB binding site in motifs in this cluster relative to the same background set of genes was confirmed using oPOSUM/JASPER [33] (data not shown).

### Pathway enrichment analysis

**Figure 7:**
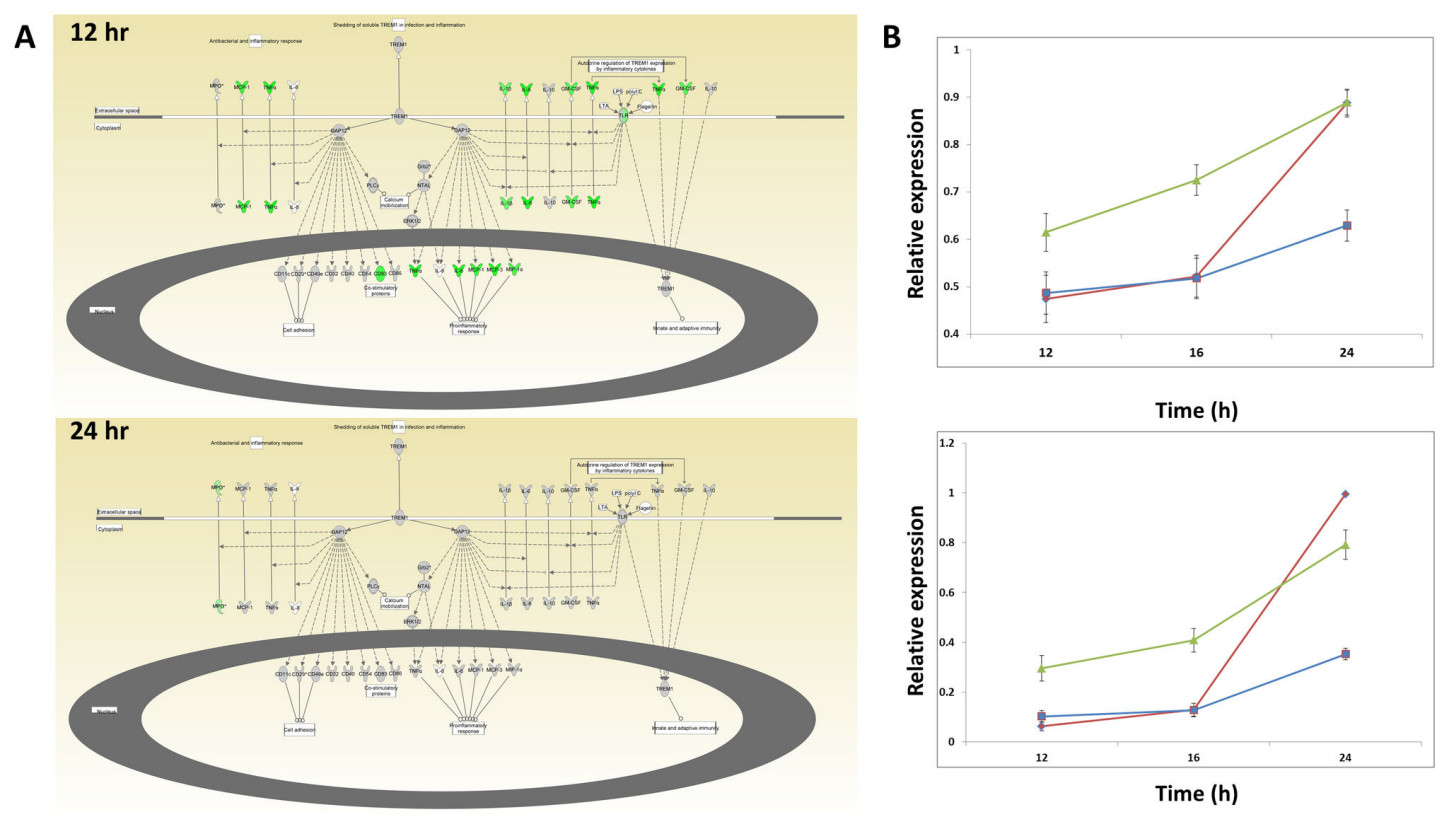
Pathway enrichment analysis. **A**) Network diagram of TREM1 signaling canonical pathway from IPA. Colored nodes indicate genes with RNA expression measures above background in murine lungs infected with influenza virus. Green indicates higher expression in X31-infection than PR-infection at the indicated time point (>= 2 Fold). Grey color indicates that the difference in expression is < 2 fold. **B**) Average expression measure of all genes above background annotated as belonging to the “TREM1 signaling” (top) and “Differential Regulation of Cytokine Production in Macrophages and T Helper Cells by IL-17A and IL-17F” (bottom) pathways. PR8=red, VN=blue, X31=green. Error bars represent the SEM.

IPA (Ingenuity Pathway Analysis) was used to identify pathways that were statistically over-represented in each cluster of genes (Table 1). Cluster 2 was of particular interest as it seemed that the genes in this cluster, which are up-regulated earlier in response to infection with the low-pathogenicity strain (X31), might constitute a protective program that limits viral replication (Figure 3 C). Notably, this cluster of genes is uniquely enriched for nine pathways, all of which are related to chemokine, NF-κB, IL-17A, and TREM1 signaling. For instance, a number of genes in the TREM1 signaling pathway show higher expression in the X31 group compared to PR8 at the 12 hour time-point (green color = higher in X31, red – higher in PR8) but, by 24 hours, those levels are roughly matched (Figure 7A). This signature of early activation by X31-infection was broadly evident across the full set of genes in the IL-17 and TREM1 pathway. Indeed, the average expression measure of all expressed genes in each of these pathways, regardless of the degree of induction, followed the average expression pattern for cluster 2 (Figure 7B). In concordance with these results, IPA analysis of the 25 genes (from the 96-gene real-time PCR panel) that were induced more rapidly by X31 (Table 2) showed enrichment profiles comparable to those found for the X31-early set defined by the array analysis (cluster 2)

**Table 1:** Ingenuity Canonical Pathways enriched in at least one expression cluster.

**Ingenuity Canonical Pathway^a^**	**C2** ^b^	**C3**	**C4**
Differential Regulation of Cytokine Production in Intestinal Epithelial Cells by IL-17A and IL-17F	3.47E-08		
Communication between Innate and Adaptive Immune Cells	6.17E-08		
TREM1 Signaling	1.1E-07		
Differential Regulation of Cytokine Production in Macrophages and T Helper Cells by IL-17A and IL-17F	2.09E-07		
Role of Cytokines in Mediating Communication between Immune Cells	0.000331		
MSP-RON Signaling Pathway	0.000331		
Dendritic Cell Maturation	0.000427		
Acute Phase Response Signaling	0.002399		
Crosstalk between Dendritic Cells and Natural Killer Cells	0.002455		
IL-17A Signaling in Fibroblasts	0.002754		
Chemokine Signaling	0.012303		
Glucocorticoid Receptor Signaling	0.016218		
NF-kB Signaling	0.016982		
LXR/RXR Activation	0.032359		
p38 MAPK Signaling	0.033884		
IL-6 Signaling	0.038019		
IL-12 Signaling and Production in Macrophages	0.038905		
G-Protein Coupled Receptor Signaling		0.006166	
Role of PI3K/AKT Signaling in the Pathogenesis of Influenza		0.019055	
Crosstalk between Dendritic Cells and Natural Killer Cells		0.039811	
Activation of IRF by Cytosolic Pattern Recognition Receptors			1.58E-11
Interferon Signaling			1.23E-10
Role of RIG1-like Receptors in Antiviral Innate Immunity			2.24E-05
Retinoic acid Mediated Apoptosis Signaling			0.000148
Role of PKR in Interferon Induction and Antiviral Response			0.000832
Role of JAK1, JAK2 and TYK2 in Interferon Signaling			0.002884
Role of MAPK Signaling in the Pathogenesis of Influenza			0.032359
IL-10 Signaling	0.012303	0.00537	
Role of Pattern Recognition Receptors in Recognition of Bacteria and Viruses	3.31E-05		3.24E-08
Role of IL-17F in Allergic Inflammatory Airway Diseases	0.000257		0.010965
Role of Hypercytokinemia/hyperchemokinemia in the Pathogenesis of Influenza	0.000132	0.000891	0.006918

^a^ IPA canonical pathways that are significantly enriched (Benjamini-Hochberg corrected p-value <= 0.05) in at least one expression cluster. Pathways containing less than three genes from the cluster or pathways not biologically relevant to lung tissue are excluded.

^b^ P-values are listed only for the expression clusters in which the pathway was enriched.

**Table 2:** Ingenuity Canonical Pathways enriched in the VN infected mTEC cultures as measured by microfluidics qPCR.

Ingenuity Canonical Pathways^a^	B-H p-value^b^	p Value	Molecules
Activation of IRF by Cytosolic Pattern Recognition Receptors	8.91E-07	1.91E-08	IRF7, STAT2, IRF9, STAT1, ISG15
Interferon Signaling	3.09E-06	1.32E-07	MX1, STAT2, IRF9, STAT1

^a^ genes induced more rapidly by VN in the mTEC cultures relative to PR and X31. Pathways containing less than three genes from the cluster or pathways not biologically relevant to trachea were excluded.

^b^ The B-H p-values indicate the significance of enrichment. P-values are listed only for the expression clusters in which the pathway was enriched.

### Tnf treatment reduces viral replication rate *in vitro*

**Figure 8:**
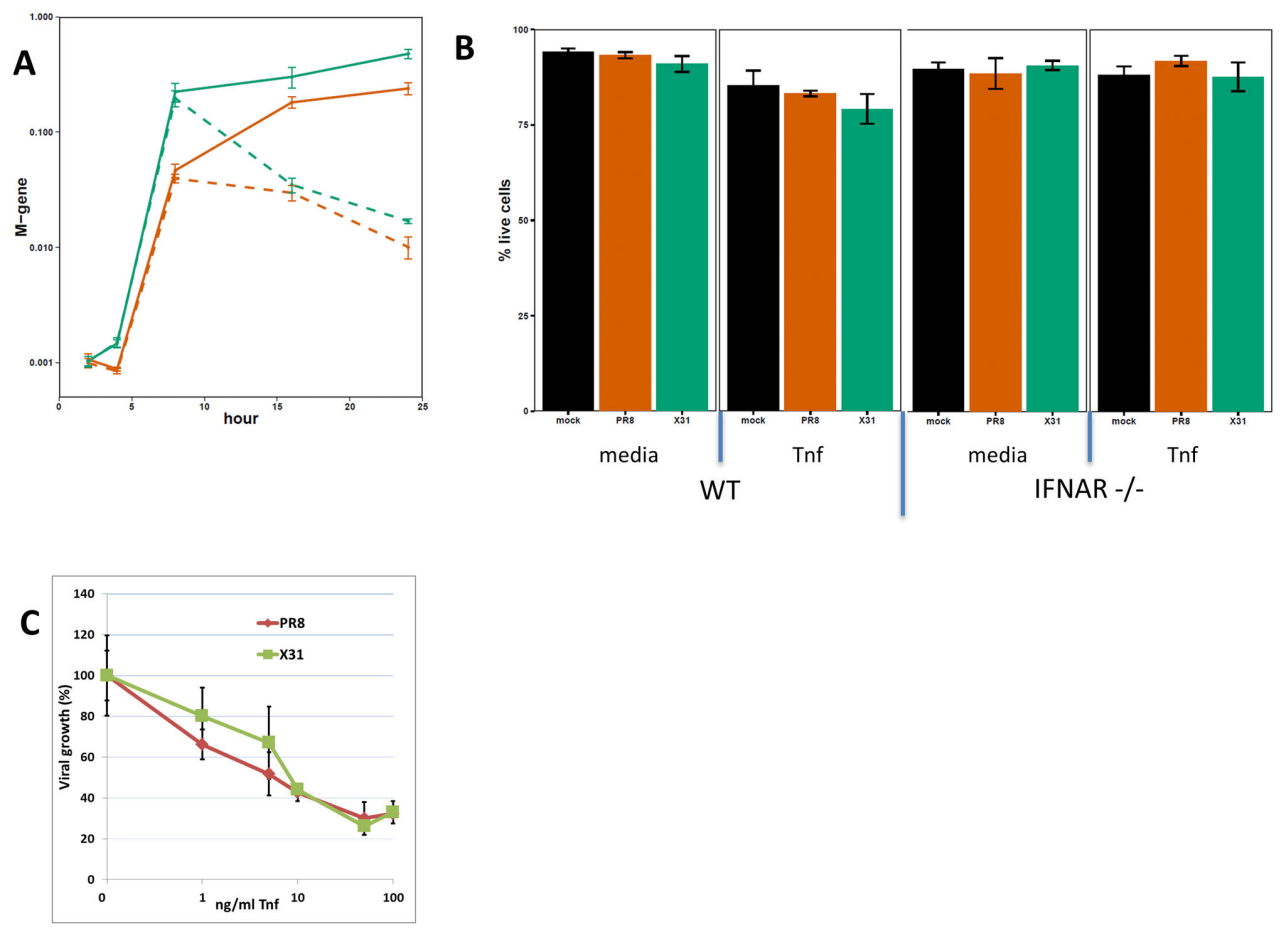
PR8 and X31 replication in LET-1 cells. A) LET-1 cells were infected with either PR8 (red) or X31 (green) and treated with TNF (100 ng/mL) two hours post infection (dashed lines) or buffer only (solid lines). (B) Percent of live LET-1 cells (derived from WT or IFNAR KO mice) at 24h post infection with or without Tnf added to the media (at 2h). (C) Tnf dose response in PR8 and X31 infected LET-1 cells (n=3). Tnf was added 2h post-infection, viral M gene was measured at 24h post-infection. Error bars represent SEM.

**Figure 9:**
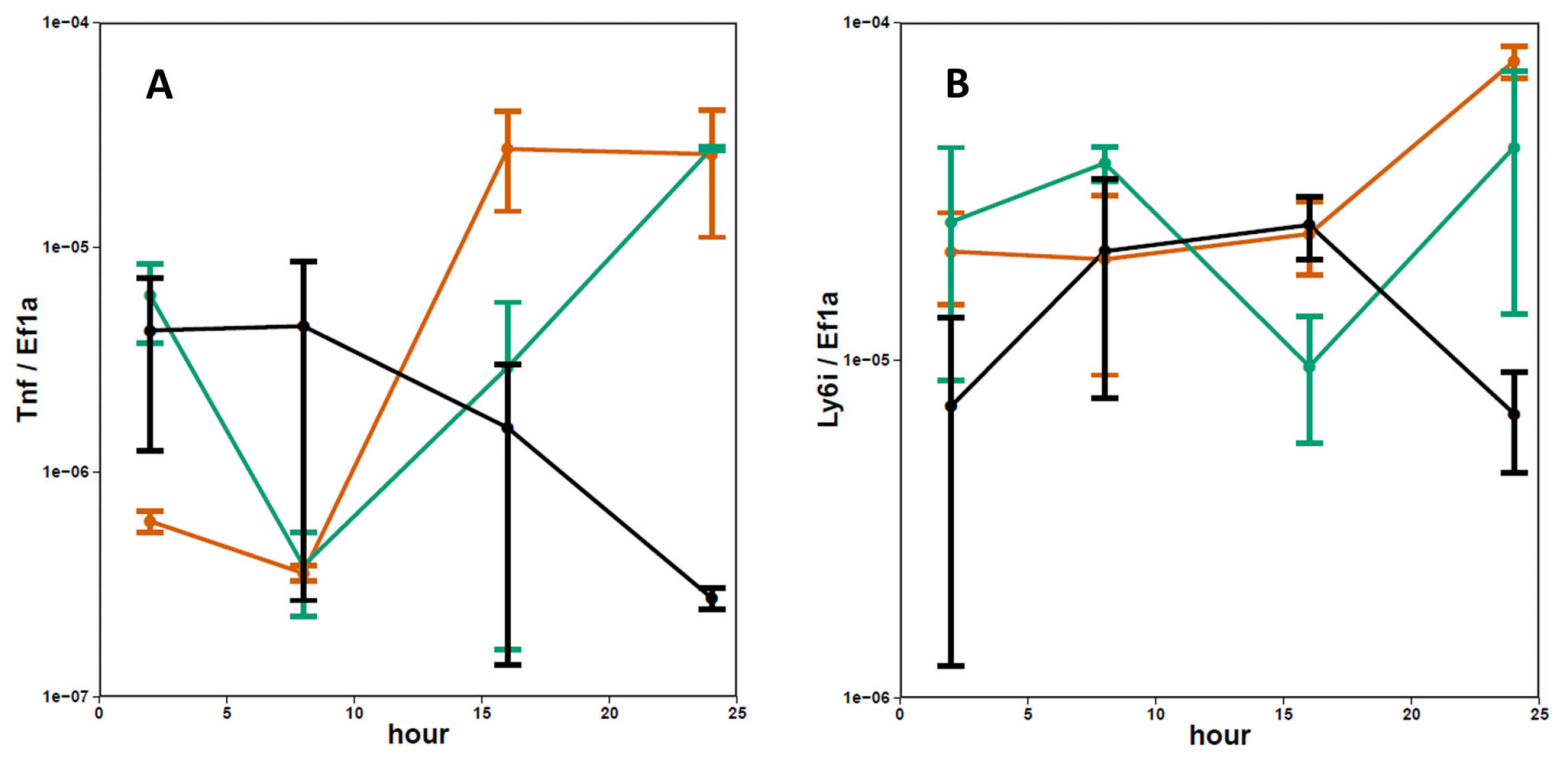
Tnf and Ly6i expression in PR8 or X31 infected LET-1 cells. LET-1 cells were infected with either PR8 (red) or X31 (green) and relative levels of endogenous (**A**) Tnf and (**B**) Ly6i transcripts determined from RNA isolated at specified time points. Error bars represent SEM for n=2.

The dramatic reduction in the replication rate of X31 *in vivo* correlates temporally with the increased expression of a number of transcripts, defined by cluster 2. One of the gene products in that cluster, Tnf, has been shown previously to reduce replication rates for influenza A viruses in lung epithelial cells [40]. Since most of the differences in viral abundance at 16-24 hours post infection reflect growth profiles in the alveolar epithelium, a type I alveolar epithelial cell line (LET-1 [C. Rosenberger, manuscript in preparation]) was used to test the effect of Tnf in this system. Though previous reports showed that pre-treatment of cells with Tnf leads to reduced production of virus [40], Tnf is normally induced subsequent to infection. We tested whether Tnf, when administered post-infection, is equally effective at reducing the growth rate of both of the strains used in this study. LET-1 cells were infected at low MOI (0.01 for PR8 and 0.05 for X31 to normalize for the M gene levels at 2 hours) and the infection was allowed to proceed with or without the addition of recombinant Tnf (100 ng/mL) added 2 hours following infection. Influenza virus growth at 24 hours post-infection was then assessed by measuring levels of the viral M gene. As shown in Figure 8 A, treating with Tnf after infection drastically reduced the levels of both PR8 and X31 RNA measured at 16 and 24 hours. To test whether this effect could be due to Tnf-induced cell death, the percent of the live cells in the cultures was determined at 24 hours following infection. Tnf treatment reduced the percentage of live cells by 9-11% in all groups (Mock, X31 and PR8), although this change was statistically significant only in PR8 group (9% -p Value 0.0009) (Figure 8 B). Given that the observed viral abundance is over 10-fold lower in Tnf treated wells, we do not consider the small differences in cell viability due to Tnf treatment to be a confounding factor in these measurements. Furthermore, Let-1 cells derived from IFNAR KO mice, showed a similar reduction in viral growth then treated with recombinant Tnf (data not shown) and no difference in cell viability (Figure 8 B). Finally, both X31 and PR8 strains show similar sensitivity to Tnf. Levels of viral RNA were equally reduced in presence of Tnf, at concentrations between 1 and 100 nm/mL (Figure 8 C - all p Values > 0.05). Without Tnf treatment, X31 and PR8 had similar replication rates in LET-1 cells, at MOIs ranging from 0.01 to 1 (Figure 8 A and data not shown) and induce similar levels of Tnf and Ly6i mRNA (Figure 9 A and B).

### Early transcriptional response in the trachea

As in the lung, all three strains replicated identically in the trachea over the first 8 hours, though their growth rates diverged sharply beyond that time point. While X31 showed reduced replication rates in the lungs (relative to PR8 and VN) from 12-48 hours, the X31 load in the tracheas of the same animals continued to rise until 24 hours. In contrast, while the lung VN RNA increased continuously over the first 48 hours, the levels in the trachea remained constant after 8 hours (Figure 3). By 24 hours, these differences in growth rates resulted in nearly 50-fold higher levels of PR8 and X31 in the trachea compared to VN. In addition, viral titers of the VN strain in the trachea were drastically lower than those of two other strains (Figure 3F).

**Figure 10:**
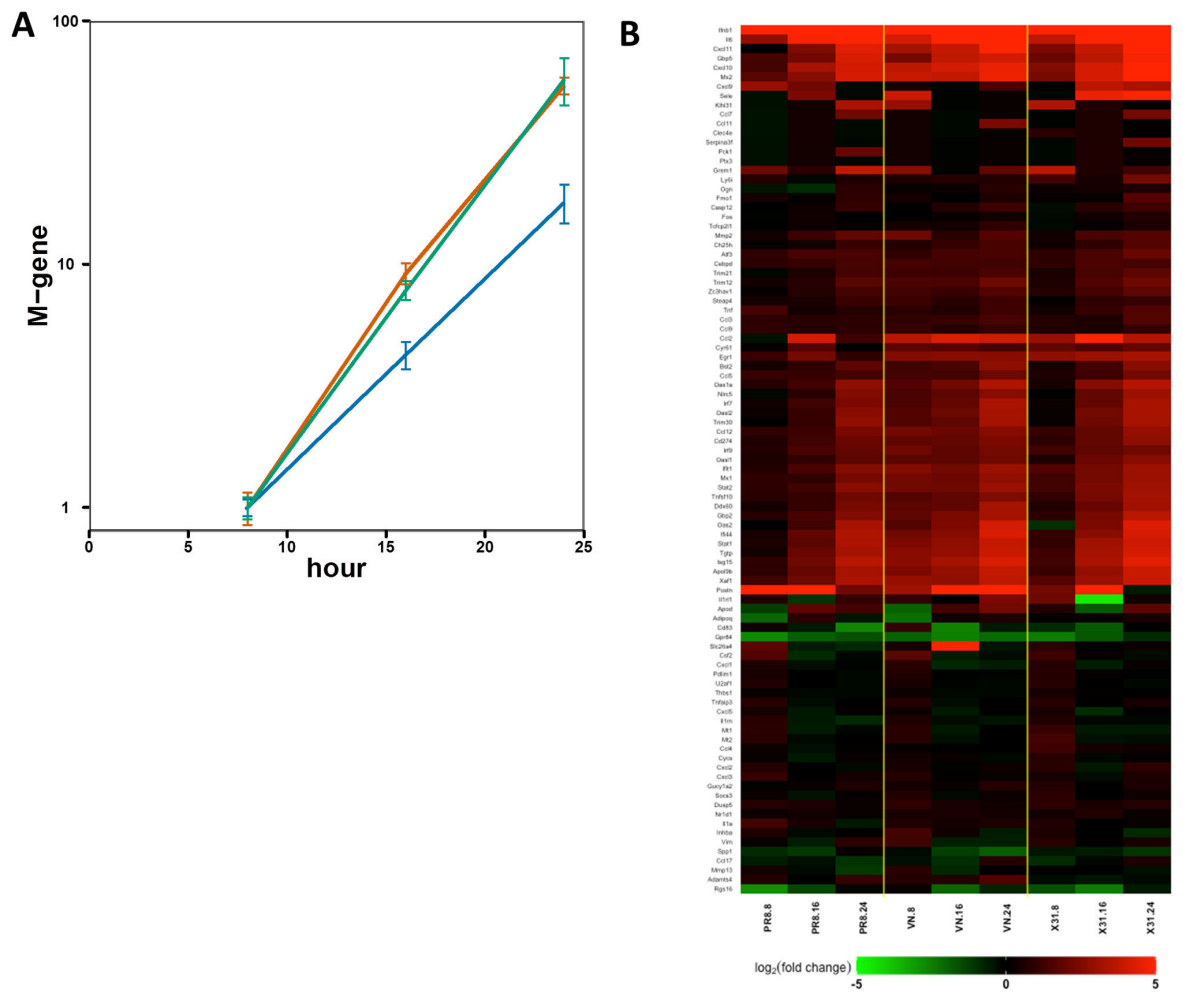
Transcriptional response to influenza infection in mTECs. **A**) Expression of the influenza M-gene in mTEC cultures infected with PR8 (red), X31 (green), or VN (blue), at an MOI of 0.01, normalized to expression at 8 hours for each strain. Values show average of 3 replicates and error bars represent the SEM. **B**) Heatmap depicting expression of 96 genes in mTEC cultures infected with X31, PR8, or VN 8-24h post-infection.

In order to identify host-responses that might contribute to the specific growth restriction of VN in the trachea, we measured the expression of the 96-gene panel described above by multiplexed real-time PCR. Unfortunately, the overall level of response in tracheas taken directly *ex vivo* prior to 8-hours was too low to be reliably measured, so we turned instead to the *in vitro* mouse tracheal epithelial cell culture (mTEC) model [30]. The mTEC cultures were infected with each of the influenza strains (PR8, X31 or VN) at low MOI (0.01) to allow a spreading infection, and virus growth kinetics were assessed by measuring levels of the M-gene. In this system, as in the tracheas taken directly from infected mice, growth of the VN strain was reduced (relative to x31 and PR8) over the first 24 hours (Figure 10 A).

The host response profiles of infected mTEC cultures were measured using the BioMark real-time qPCR platform and the 96-gene panel (rather than by microarrays) in order to maximize sensitivity for differential expression. A total of 24 transcripts were identified (Figure 10 B) where induction was higher at 8 hours after infection with VN relative to both X31 and PR8 (p < 0.05). Remarkably, within this 96-gene panel, there was no overlap between the set of genes induced early by VN in mTECs and at comparable times by X31 in the lungs. IPA analysis of these 24 genes showed only two pathways to be significantly enriched, “Activation of IRF by cytosolic PRRs” and “IFN signaling” (Table 2), neither of which features in cluster 2 from the microarray analysis of whole lung (Table 1) or in the 25 genes from the 96-gene panel that were induced more strongly (p < 0.05) in the lung at either 12 or 16 hours following x31 infection (Table 3). Overall, the implication is that different mechanisms may operate to restrict virus replication in these two tissues.

**Table 3:** Ingenuity Canonical Pathways enriched in the lungs of X31 infected mice as measured by microfluidics qPCR.

Ingenuity Canonical Pathways ^a^	B-H p-value^b^	pValue	Molecules
Communication between Innate and Adaptive Immune Cells	3.16228E-12	3.16228E-14	IL1A, CCL4, IL1RN,CCL3L1/CCL3L3,CD83,CCL3,CSF2,TNF,Ccl9
Differential Regulation of Cytokine Production in Intestinal Epithelial Cells by IL-17A and IL-17F	1E-10	1.99526E-12	IL1A, CCL4, CCL2, CCL3, CSF2, TNF
Role of Hypercytokinemia/hyperchemokinemia in the Pathogenesis of Influenza	2.69153E-09	1E-10	IL1A, CCL4, CCL2, IL1RN,CCL3,TNF
Differential Regulation of Cytokine Production in Macrophages and T Helper Cells by IL-17A and IL-17F	2.69153E-09	1.12202E-10	CCL4, CCL2, CCL3, CSF2, TNF
TREM1 Signaling	1.44544E-08	7.58578E-10	CXCL3, CCL2, CD83, CCL3, CSF2, TNF
Glucocorticoid Receptor Signaling	4.46684E-06	2.81838E-07	CXCL3, CCL2, IL1RN,CCL3,CSF2,CCL11,TNF
Role of Cytokines in Mediating Communication between Immune Cells	2.23872E-05	2.39883E-06	IL1A, IL1RN,CSF2,TNF
IL-10 Signaling	5.12861E-05	6.45654E-06	SOCS3, IL1A, IL1RN,TNF
Dendritic Cell Maturation	9.77237E-05	1.31826E-05	IL1A, IL1RN,CD83,CSF2,TNF
IL-6 Signaling	0.000316228	5.37032E-05	SOCS3, IL1A, IL1RN,TNF
Role of IL-17F in Allergic Inflammatory Airway Diseases	0.000323594	5.88844E-05	CCL4, CCL2, CSF2
LXR/RXR Activation	0.000338844	6.30957E-05	IL1A, CCL2, IL1RN,TNF
Acute Phase Response Signaling	0.001	0.000218776	SOCS3, IL1A, IL1RN,TNF
Chemokine Signaling	0.001122018	0.00025704	CCL4, CCL2, CCL11
FXR/RXR Activation	0.001949845	0.000489779	IL1A, IL1RN,TNF
Crosstalk between Dendritic Cells and Natural Killer Cells	0.001949845	0.000489779	CD83, CSF2, TNF
HMGB1 Signaling	0.002344229	0.000616595	IL1A, CCL2, TNF
PPAR Signaling	0.002344229	0.000645654	IL1A, IL1RN,TNF
p38 MAPK Signaling	0.003630781	0.001071519	IL1A, IL1RN,TNF
NF-kB Signaling	0.01	0.003311311	IL1A, IL1RN,TNF

^a^ genes induced more rapidly by X31 in the lung relative to VN and PR8. Pathways containing less than three genes from the cluster or pathways not biologically relevant to lung were excluded.

^b^ The B-H p-values indicate the significance of enrichment. P-values are listed only for the expression clusters in which the pathway was enriched.

## Discussion

In this study, we measured in detail the very early replication rates in mouse lungs and tracheas of three strains of influenza with varying pathogenicity. Remarkably, no differences in replication rate or viral abundance were observed for the first 16 hours in the lung and 8 hours in the trachea (Figure 3 A-D). After those time points, RNA levels of X31 in the lungs and VN in the trachea started to significantly lag behind the other two strains. These measurements were concordant with histological scoring of lung sections which consistently showed higher levels of PR8 and VN at 24 hours (Figure 2B) as well as viral titer measurements in both tissues (Figure 3E and F).

Measuring the global transcriptional response in mouse lung and tracheal cells over the first 48 hours following infection with three influenza viruses of varying pathogenicity has allowed us to identify a set of genes and pathways in these tissues where accelerated activation by specific strains correlates with reduced virus growth. To the best of our knowledge, this is the most detailed transcriptional analysis to of early influenza virus infection in a whole animal context.

Innate immune responses in airway epithelial cells provide the first line of defense by directly limiting influenza virus growth in infected epithelium and by rendering neighboring cells less amenable to infection, primarily via the secretion of type I and III interferons. Numerous studies have shown that ablation of individual innate immune genes generally results in enhanced influenza virus replication [20,21,41-44] while complete loss of type I and III interferon responses leads to unrestrained virus growth and rapid death [19]. Even so, while the innate immune system plays a critical role in the first days of infection, a functioning adaptive immune response is still necessary for the resolution of the influenza virus infection [45]. It is likely that the extent of infection at the time the adaptive response initiates can have a profound influence on disease outcome. A greater number of infected cells and higher levels of virus would, for example, be expected to result in more tissue damage following the influx of antigen-specific cytotoxic T cells.

The host response to the infection was very robust even at early time points. Using ANOVA and very stringent cutoffs, we have identified 385 genes whose expression changed in the first 24 hours. Of those genes, 351 were up-regulated and many of them are known to respond to type I interferons (Gbp1, Gbp2, Gbp5, Gbp6, Gbp10, Ifi204, Ifi203, etc.). Not surprisingly, Ingenuity Pathway Analysis identified a number of proinflammatory pathways enriched in this dataset (Innate immune response, Interferon and cytokine signaling, IRF activation by PRRs, etc.). A number of those genes have been shown previously to play a protective role during the influenza infection (e.g. Isg20, Isg15, Gbp1, etc. [17,46,47]).

In the lungs, all three strains (PR8, X31, and VN) replicate equally rapidly for the first 12 hours following infection, representing approximately two viral life-cycles [48]. This suggests that there are minimal intrinsic differences between the inherent capacities of these viruses to replicate in murine lung cells *in vivo*. After 12 hours, however, the growth of X31 is significantly retarded relative to the other two strains, and an additional 12 hours elapses before the growth rates of the high pathogenicity PR8 and VN viruses are similarly restricted. The net consequence is 5- to 7-fold higher levels of VN and PR8 in the lung (relative to X31) by 48 hours after infection. Although in some experiments, M gene levels of the PR8 virus inoculum were higher than those of the other two strains (Figure 3, 0-hour time point and data now shown) those differences were not found to be consistent. This is not surprising given that in most experiments the measured levels at early time points were low and the experimental uncertainty is high. Over the next few hours, viral RNA levels converge and show no differences up to 16 hours post-infection. In contrast, X31 M gene levels show clear differences compared to the other two strains at 24 hours and later time points in the lungs.

Most of the differences in gene expression changes between infections with different strains did not correlate with the disease phenotype (clusters 1, 3, 4 and 5 in the Figure 4) and were not the focus of this paper. In contrast, genes in cluster 2 show increased expression in the X31 infected animals as early as 8 hours (when the viral loads are the same for all three strains) and the expression levels of these genes do not reach equivalent levels until 24 hours following PR8- and VN-infection. This cluster is strongly enriched for genes in the TREM1 and IL-17 signaling pathways, implicating these signaling cascades in the control of influenza virus replication. Considering that both these pathways have been shown to require NF-kB signaling [49-52], it was not surprising that promoter analysis of the cluster 2 genes showed very significant enrichment for the NF-kB binding sites. It is also possible that the observed changes in expression for the genes in cluster 2 are caused by (rather than a cause of) differential replication rates. However, considering that the differences in host response occur first (as early as 8 hours), we consider it more likely that the host response leads to the observed differences in viral load levels at 24 hours and later.

The TREM1 and IL-17 pathways are both pro-inflammatory and have been shown to play significant and complex roles in the control of both bacterial and viral infections. Silencing of TREM1 activation *in vivo* increased mortality in a mouse model of bacterial peritonitis while partial silencing enhanced survival [53]. Genes belonging to the TREM1 signaling pathway were found to be differentially regulated in the mouse lungs, most likely in infiltrating neutrophils, during the infection by the high and low virulence H3N2 viruses [54]. Furthermore, IL-17 signaling has been shown to synergistically enhance the expression of pro-inflammatory genes without affecting the IFN-stimulated genes [55]. Also, following i.n. challenge with PR8, IL-17RA^-/-^ mice show reduced levels of the oxidized phospholipids that are critical mediators of acute lung inflammation, and have better survival rates than the wild type controls [56]. IL-17 also has been shown to play a role in lung damage in mice infected with the 2009 swine-origin H1N1 pandemic virus [57]. Overall, while excessive activation of the TREM1 and IL17 signaling pathways at later time points may be detrimental to the host, our results show that the early activation of this pathway correlates with increased protection against influenza virus challenge. This possibility should be considered when exploring anti-inflammatory therapies targeting these two signaling pathways.

Because the attenuated growth of X31 (relative to PR8 and VN) was only observed *in vivo*, it is likely that strain-specific differences in infectivity and tropism for the various cell types in the lung contribute to this effect. The expression analysis presented here is for whole, virus infected lung tissue, so it is possible that the apparent early activation of the TREM1 and IL-17 signaling pathways by X31 arises from differences in the inflammatory cell populations recruited to the lung at these time points or simply from different resident cells being infected. Indeed, some of the transcripts from cluster 2 (genes induced more rapidly by X31) are known to be expressed in immune cells (Il1b, Nod2, Tnfaip2, Casp4, Mcoln2, Ccl3, Gbp5, Pim1 and Ccl4). It has been reported previously that the infectability of various cell types by influenza virus is sub-type dependent. For instance, the H3N2 strains Bjx109 and X31 were found to infect macrophages to much higher levels than the H1N1 strain PR8 [58,59]. We are currently exploring this possibility by isolating specific cell populations from the infected lung, measuring their relative levels of infection, and determining the contribution of these different inflammatory cell subsets to the overall transcriptional response found here for virus-infected lung tissue.

The role of the Tnf in the pathogenesis of influenza is poorly understood. While *in vitro* analysis in cell culture systems clearly demonstrates that pre- or post-treatment with recombinant Tnf can reduce influenza virus replication rates (Figure 8) [40], the role of Tnf *in vivo* is less clear. Studies in mice lacking Tnf signaling (TNFR1^-/-^) showed slightly delayed morbidity, but little or no difference in the mortality associated with influenza virus infection [23,60,61]. Interestingly, virus titers at 24 hours after infection did not differ in the lungs of WT and TNFR1^-/-^ mice [23]. A more recent study found that the lack of Tnf signaling in mice led to increased tissue damage (possibly mediated via MCP-1) during influenza virus infection, even though there was no ultimate difference in clinical outcome [62]. Finally, during the 2009 influenza pandemic, several Tnf associated SNPs identified in human patients were found to be highly correlated with disease severity [63]. Our results show that the replication rates of both X31 and PR8 in the LET-1 cells can be similarly reduced by recombinant Tnf, suggesting that the early induction of Tnf by X31 might contribute to its reduced replication rate *in vivo*. This could offer a plausible explanation on how a less pathogenic influenza virus is controlled earlier in the lungs of infected mice although, at this stage, it is not clear what triggers the Tnf response, or which cells are responsible for its production.

Differences in the virus growth and host response profiles observed in the lungs of infected animals were not consistently replicated in cultures of those same cell types. While the growth profiles for PR8 and X31 determined *in vivo* for type I epithelial cells show different kinetics (Figure 2 C), these two strains replicate similarly in monolayers of the LET-1 cell line (Figure 8A – solid lines). Both strains also showed comparable growth characteristics and triggered indistinguishable host response in other culture systems (data not shown), including a Type II epithelial cell line (MLE-12), an alveolar macrophage cell line (MH-S), and a dendritic cell line (JAWSII) highlighting the difficulty of translating findings about influenza virus pathogenicity from *in vitro* to whole animal systems. In addition, the host response observed in the *in vitro* system is clearly different than that observed in the *in vivo* experiments. For example, two genes, Tnf and Ly6i that show strong X31 specific induction in lungs relative to PR8, respond identically in LET-1 cells (Figure 9 A and B). These data suggest that the observed *in vivo* host response and outcome likely arises from the complex interplay of several cell types.

Another H5N1 virus (mouse adapted A/crested_eagle/Belgium/1/2004) has, like VN, also been shown to replicate poorly in the murine upper respiratory tract while replicating aggressively in the lung [64]. The reduced growth rate of the VN strain in the mTEC system correlated with the activation of classic IRF-driven interferon signaling which occurred several hours earlier than in PR8 or X31 groups, suggesting that type I interferon responses may play a prominent role in the early control of influenza virus replication in the trachea. Given that the viruses used in this study differ in their HA and NA genes, it is possible that in both cases, differences in host response reflect infection of different cell types (tropism). Although we did not measure infectivity of progeny virions isolated directly from the trachea, experiments using PR8, X31, and VN in mTEC cultures indicated that all three strains of virus produced in mTECs can infect new mTEC cultures (data not shown). In addition, when mTECs are infected at high MOI, influenza M gene levels are approximately the same at 12 hours for all three viruses (data not shown). The differences for VN levels relative to the other two strains, were only observed at low MOI (in trachea and mTECs) – in a situation where the capacity for virus spread is a key feature. Taken together, these data are suggestive that the arrested growth of the VN strain in the trachea relative to PR8 and X31 likely arises from earlier activation of the interferon response rather than from any inherent inability to replicate infectious virions.

Thus, in this mouse model, influenza virus pathogenicity is determined by differential lung infection profiles. Similarly, influenza virus infection of the human lung, as opposed to the upper airway, is generally associated with more severe disease. The presented mouse experiments further suggest that host fate can be determined within hours of infection by the differential activation (in target lung tissue) of genes in the IL-17 and TREM1 pathways. These observations support the view that the early, regulated production of downstream targets of the IL-17 and TREM1cascade is protective, while over-exuberant activation at later times after infection may well be detrimental.
